# Olive Oil effectively mitigates ovariectomy-induced osteoporosis in rats

**DOI:** 10.1186/1472-6882-11-10

**Published:** 2011-02-04

**Authors:** Nermine K Saleh, Hanan A Saleh

**Affiliations:** 1 Faculty of Medicine, Ain Shams University, Physiology Department, Egypt; 2 Faculty of Medicine, Ain Shams University, Histology Department, Egypt

## Abstract

**Background:**

Osteoporosis, a reduction in bone mineral density, represents the most common metabolic bone disease. Postmenopausal women are particularly susceptible to osteoporosis when their production of estrogen declines. For these women, fracture is a leading cause of morbidity and mortality. This study was conducted to evaluate the protective effects of olive oil supplementation against osteoporosis in ovariectomized (OVX) rats.

**Methods:**

We studied adult female Wistar rats aged 12-14 months, divided into three groups: sham-operated control (SHAM), ovariectomized (OVX), and ovariectomized rats supplemented with extravirgin olive oil (Olive-OVX) orally for 12 weeks; 4 weeks before ovariectomy and 8 weeks after. At the end of the experiment, blood samples were collected. Plasma levels of calcium, phosphorus, alkaline phosphatase (ALP), malondialdehyde (MDA), and nitrates were assayed. Specimens from both the tibia and the liver were processed for light microscopic examination. Histomorphometric analysis of the tibia was also performed.

**Results:**

The OVX-rats showed a significant decrease in plasma calcium levels, and a significant increase in plasma ALP, MDA, and nitrates levels. These changes were attenuated by olive oil supplementation in the Olive-OVX rats. Light microscopic examination of the tibia of the OVX rats revealed a significant decrease in the cortical bone thickness (CBT) and the trabecular bone thickness (TBT). In addition, there was a significant increase in the osteoclast number denoting bone resorption. In the Olive-OVX rats these parameters were markedly improved as compared to the OVX group. Examination of the liver specimens revealed mononuclear cellular infiltration in the portal areas in the OVX-rats which was not detected in the Olive-OVX rats.

**Conclusions:**

Olive oil effectively mitigated ovariectomy-induced osteoporosis in rats, and is a promising candidate for the treatment of postmenopausal osteoporosis.

## Background

Osteoporosis represents the most common metabolic bone disease. The most common type of osteoporosis is the bone loss associated with ovarian hormone deficiency at menopause [1]. The increasing incidence of postmenopausal osteoporosis and its related fractures have become global health issues in the recent days [2]. Estrogen deficiency has been regarded as a critical cause of osteoporosis, which can result from naturally or surgically induced menopause. Several placebo-controlled trials have shown that hormone replacement therapy (HRT) prevents bone loss in women in early and late postmenopause. Observational studies have shown that HRT is associated with a 30%-50% reduction of hip, spine, and wrist fractures [3]. Although HRT has been proven efficacious in preventing bone loss, it is not desirable to many women due to its side-effects [3]. Therefore, it is necessary to search for treatment modalities that not only decrease morbidity, but also having minimal side effects.

Olive oil has been reported to favor the mineralization and development of bones [4]. It is a complex compound made of fatty acids, vitamins, volatile components and water soluble components. Olive oil is rich in monounsaturated fatty acids (mainly oleic acid). In addition, it contains adequate amounts of linoleic acid. It contains a group of related natural products with potent antioxidant properties, which are esters of tyrosol and hydroxytyrosol, including oleocanthal and oleuropein as well as vitamin E.

In light of the aforementioned data, we undertook this study to evaluate the efficacy of olive oil supplementation in preventing menopause-induced osteoporosis using the ovariectomized rat model. Recently, the importance of preventive medicine has been gradually recognized in the field of orthopaedic surgery with a concept that peak bone mass should be increased in childhood as much as possible for the prevention of osteoporosis [5]. Under such background, we have supplemented Ovariectomized (OVX) - rats with olive oil four weeks before ovariectomy with an aim to evaluate the efficacy of olive oil supplementation starting before menopause.

## Methods

This work was conducted in the Physiology Department, Faculty of Medicine, Ain Shams University, and was approved by FMASU, REC, Cairo, Egypt.

This work was undertaken on female Wistar rats aged 12-14 months. The rats were maintained under standard conditions of boarding. The rats were fed a diet constructed in our laboratory according to the normal nutritional dietary requirement (59% of food intake from carbohydrates, 7% from fat, 21% from protein, 13% from minerals and ash). The investigation conforms to the Guide for the Care and Use of Laboratory Animals published by the US National Institutes of Health. Rats were allocated into 3 groups: a) SHAM-operated control (SHAM) rats (n = 6), b) Ovariectomized (OVX) rats (n = 6), c) Olive-supplemented Ovariectomized (Olive-OVX) rats (n = 7), ovariectomized rats were orally administered with extra virgin olive oil (1 ml/100 g B.W) [6] by oral tubal feeding for 12 weeks; 4 weeks before ovariectomy and continued for 8 weeks after operation. Extra virgin olive oil was obtained from Sinai, Egypt (fatty acids composition is: 75% monounsaturates, 13% polyunsaturates and 12% saturates, and phenolic concentration is 200 mg/100 g olive oil).

Bilateral ovariectomy and sham-operation were performed under ether anesthesia. A midline longitudinal incision was made inferior to the rib cage. The ovaries of rats in groups (OVX, Olive-OVX) were exteriorized, ligated and excised. The SHAM-operated control rats had their dermal integuments, muscles and peritoneum sectioned, but underwent no excision of the ovaries.

### Experimental procedures

On the day of experiments, overnight fasted rats were weighed and injected intraperitoneally with heparin sodium, 1000 IU (B. Braun Melsungen AG.D-34209 Melsungen, Germany). One hour later, the rats were anaesthetized with thiopental sodium 40 mg/kg intraperitoneally (Sandoz GmbH, Kundl-Austria). Blood samples were collected from the abdominal aorta then centrifuged, and the plasma was subjected to the following assays: calcium, phosphorus, alkaline phosphatase (ALP), malondialdehyde (MDA), and nitrates. Tibia and liver specimens were isolated and processed for histological examination.

#### Biochemical determinations

Plasma calcium, phosphorus, and alkaline phosphatase levels were measured using standard laboratory methods.

##### Plasma MDA assay

The plasma MDA levels were determined according to the method of Draper and Hadley (1990) [7], based on the reaction of MDA with thiobarbituric acid (TBA). The reaction was performed at 95°C for 15 minutes. The sample was mixed with 2.5 volumes of 10% (w/v) trichloroacetic acid to precipitate the protein. The precipitate was pelleted by centrifugation and an aliquot of the supernatant was allowed to react with an equal volume of 0.67% TBA in a boiling water bath for 15 minutes. After cooling, the absorbance was read at 532 nm.

##### Plasma nitrate assay

Plasma nitrate levels were measured according to the method of Bories and Bories (1995) [8].

#### Histological examination of bone

The tibias were dissected out, fixed in 10% buffered neutral paraformaldehyde, and decalcified in EDTA solution for 2 weeks. Once decalcified, the specimens followed routine histological processing and were embedded in paraffin. Paraffin sections (5 μm thick) from the metaphysis of the tibias were deparaffinized and stained by haematoxylin & eosin (H&E) [9], and by Azure II- Methylene blue [10] for light microscopic examination.

#### Histological examination of liver

Liver specimens were fixed in 10% buffered neutral paraformaldehyde solution, processed and embedded in paraffin. Thin paraffin sections (5 μm) were stained by H&E [9].

#### Morphometric analysis

The mean cortical bone thickness (CBT) and the mean trabecular bone thickness (TBT) were measured in 5 fields/slide from 5 slides for each rat. The reading of each animal was considered as one variable. Also the number of osteoclasts was determined/10 HPF. The measurements were done using the image analyzer (Leica Q 500 MC program) in the Histology Department, Ain Shams University.

### Statistical analysis

Data are expressed as means ± SEM. Statistical significance for data was determined using a one-way analysis of variance *(ANOVA) *with post-hoc test, significance calculated by LSD (least significant difference) multiple range-test to find inter-group significance. The level of significance was accepted as *P *<*0.05*.

## Results

### Biochemical parameters

The results of this study clearly demonstrated significant decrease in the plasma Ca^2+ ^level 8 weeks after ovariectomy in OVX-rats as compared to both the SHAM-operated control rats as well as the Olive-OVX rats. This decrease was significantly prevented by olive oil supplementation in the Olive-OVX group.

Plasma inorganic phosphorus level showed non-significant changes among the studied groups. Plasma alkaline phosphatase level was significantly increased in the OVX-rats as compared to the SHAM-operated control rats. The administration of olive oil resulted in normal level of alkaline phosphatase as compared to the SHAM-operated control as well as to the OVX-rats (Table 1).

**Table 1:** Plasma calcium, phosphorus, and ALP activity in: SHAM-operated control (SHAM) rats, Ovariectomized (OVX) rats, and Olive-supplemented ovariectomized (Olive-OVX) rats.

	SHAM	OVX	Olive-OVX
Calcium (mg/dl)	10.87 ± 0.37 n = 6	9.5 ± 0.51 **^a, b^** n = 6	10.71 ± 0.33 n = 7
Phosphorus (mg/dl)	5.9 ± 0.28 n = 6	4.9 ± 0.47 n = 6	4.8 ± 0.31 n = 7
ALP activity (IU/liter)	144.5 ± 16.8 n = 6	202.5± 17.3 **^a^** n = 6	155.7 ± 20 n = 7

n; is the number of observations.

(a) Significance calculated by least significant difference (LSD), P < 0.05 from SHAM-operated control group.

(b) Significance calculated by LSD, P < 0.05 between OVX and Olive-OVX groups.

### Plasma malondialdehyde level

MDA was significantly increased in the OVX-rats as compared to the SHAM-operated control rats. This increase was prevented by olive oil supplementation (Table 2).

**Table 2:** Plasma MDA and Nitrates levels in: SHAM-operated control (SHAM) rats, Ovariectomized (OVX) rats, and Olive-supplemented ovariectomized (Olive-OVX) rats.

	SHAM	OVX	Olive-OVX
MDA (μmol/l)	1.18 ± 0. 14 n = 6	2.57 ± 0.40**^a^** n = 6	1.85 ± 0.24 n = 6
Nitrates (μmol/l)	15.6 ± 1.11 n = 5	21.12 ± 4.37^**b**^ n = 5	9.13 ± 3.71 n = 6

n; is the number of observations.

(a) Significance calculated by least significant difference (LSD), P < 0.05 from SHAM-operated control group.

(b) Significance calculated by LSD, P < 0.05 between OVX and Olive-OVX groups.

### Plasma nitrates level

Nitrate level was significantly increased in the OVX-rats as compared to the Olive-OVX rats (Table 2).

### Bone

Examination of sections of the SHAM-operated control group revealed that the proximal metaphysis of tibia was formed of an outer shell of compact bone (cortical bone) and inner trabeculae of cancellous bone. The compact bone consisted of outer, inner and interstitial bone lamellae as well as Haversian systems. Osteocytes resided in their lacunae inbetween the lamellae. The shell of compact bone was covered by periosteum and lined by endosteum.

**Figure 1:**
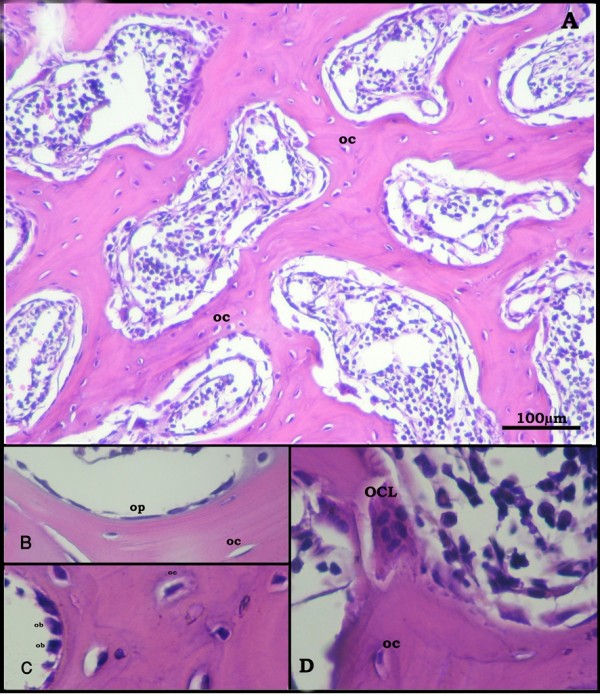
**Proximal metaphysis of tibia of the control group (H&E)**. A: a network of trabeculae of cancellous bone. Osteocytes (oc) reside in their lacunae in between the irregular bone lamellae. Bone marrow spaces are seen between the trabeculae. (×250) B: osteoprogenitor cells (op), C: Ostoblasts (ob), and D: osteoclasts (OCL) appear lining the endosteal surface. (×640)

**Figure 2:**
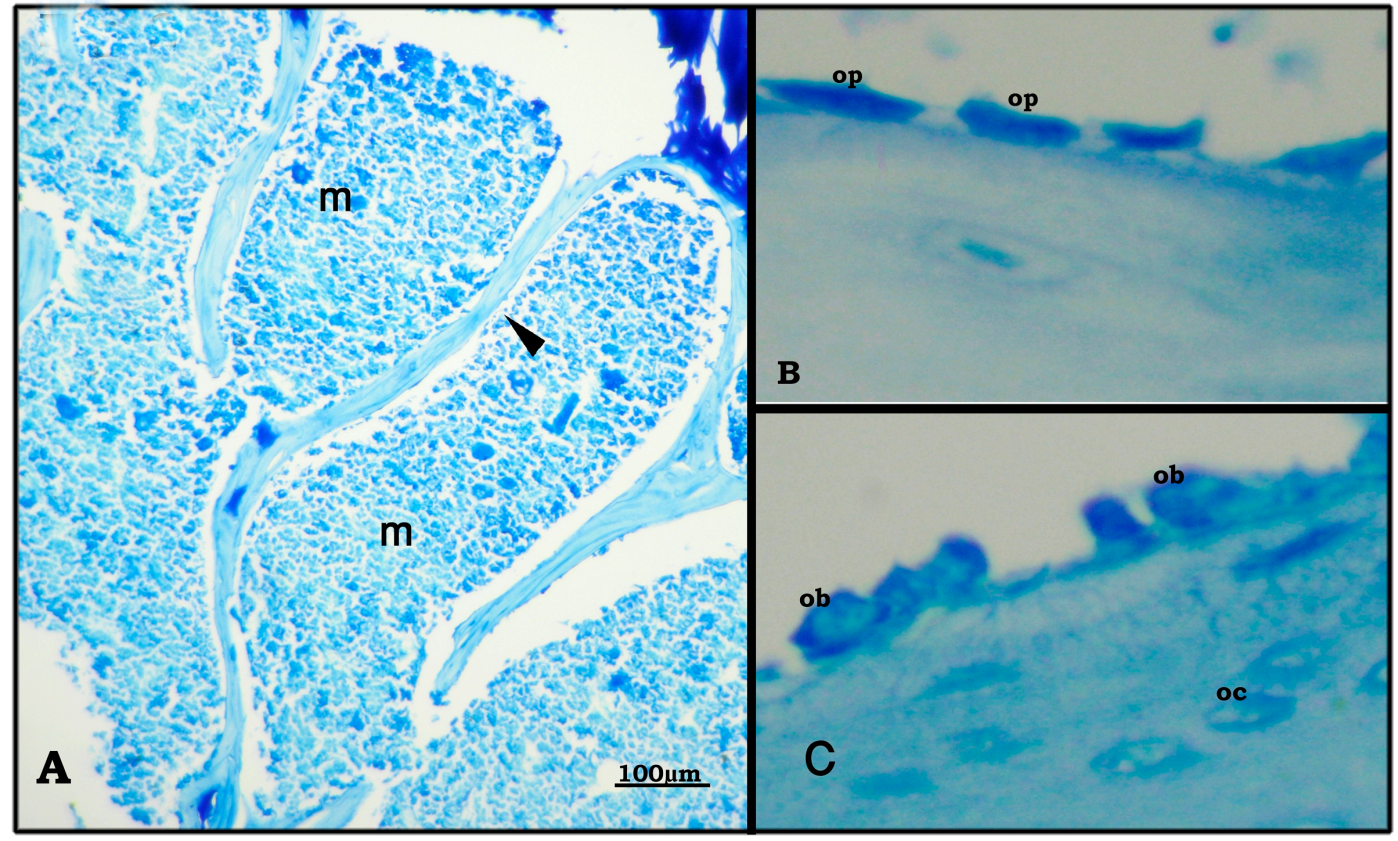
**Proximal metaphysis of tibia of the control group (Azure II-Methylene blue)**. A: bone trabeculae (black triangle) with bone marrow spaces (m) inbetween (×250). B: osteoprogenitor cells (op), and C: osteoblasts (ob) line the endosteal surface. (×640).

Cancellous bone was formed of a network of bone trabeculae composed of irregular bone lamellae between which osteocytes appeared in their lacunae (Figure 1A, 2A). The endosteal surface of trabeculae was lined by osteoprogenitor cells (Figure 1B, 2B), osteoblasts (Figure 1C, 2C) and osteoclasts in Howship's lacunae (Figure 1D). Bone marrow spaces were seen between the trabeculae (Figure 1A, 2A).

**Figure 3:**
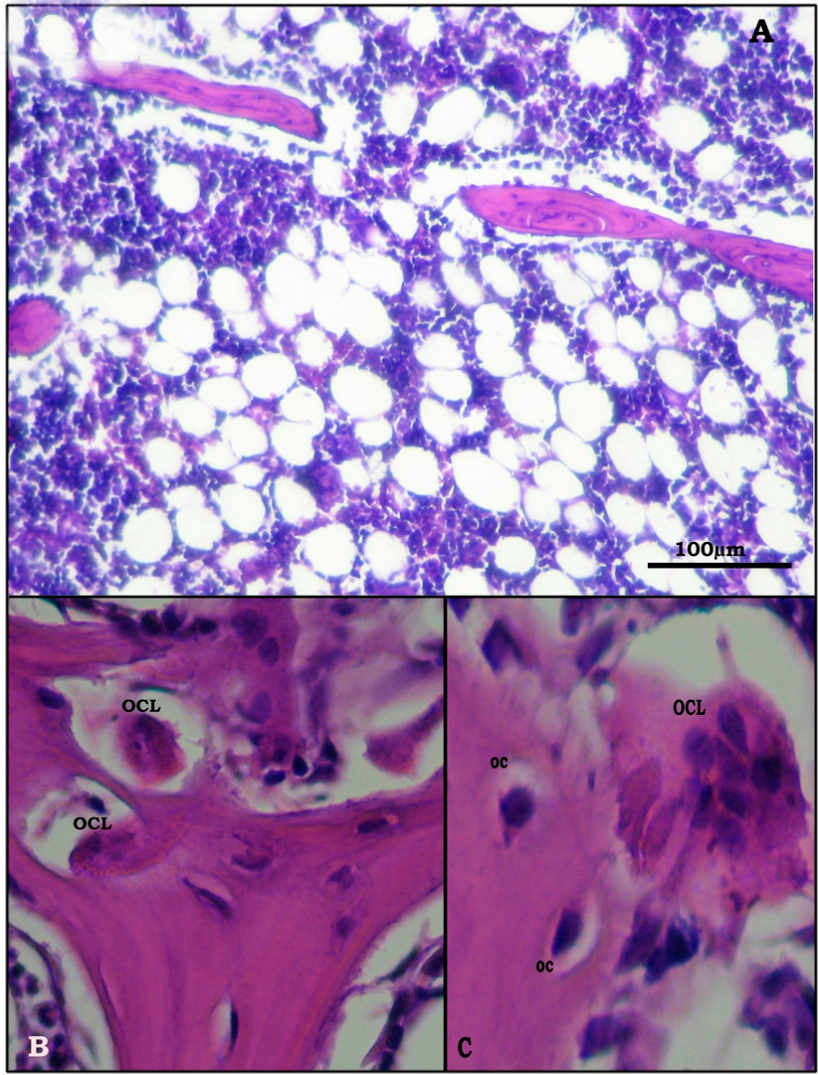
**Proximal metaphysis of tibia of the OVX-group (H&E)**. A: loss of the normal architecture of trabeculae of cancellous bone with widening of bone marrow spaces (×200). B: apparent increase in the number of osteoclasts (OCL).(×640) C: higher magnification of multinucleated osteoclast (OCL) in Howship's lacunae (×1000).

**Figure 4:**
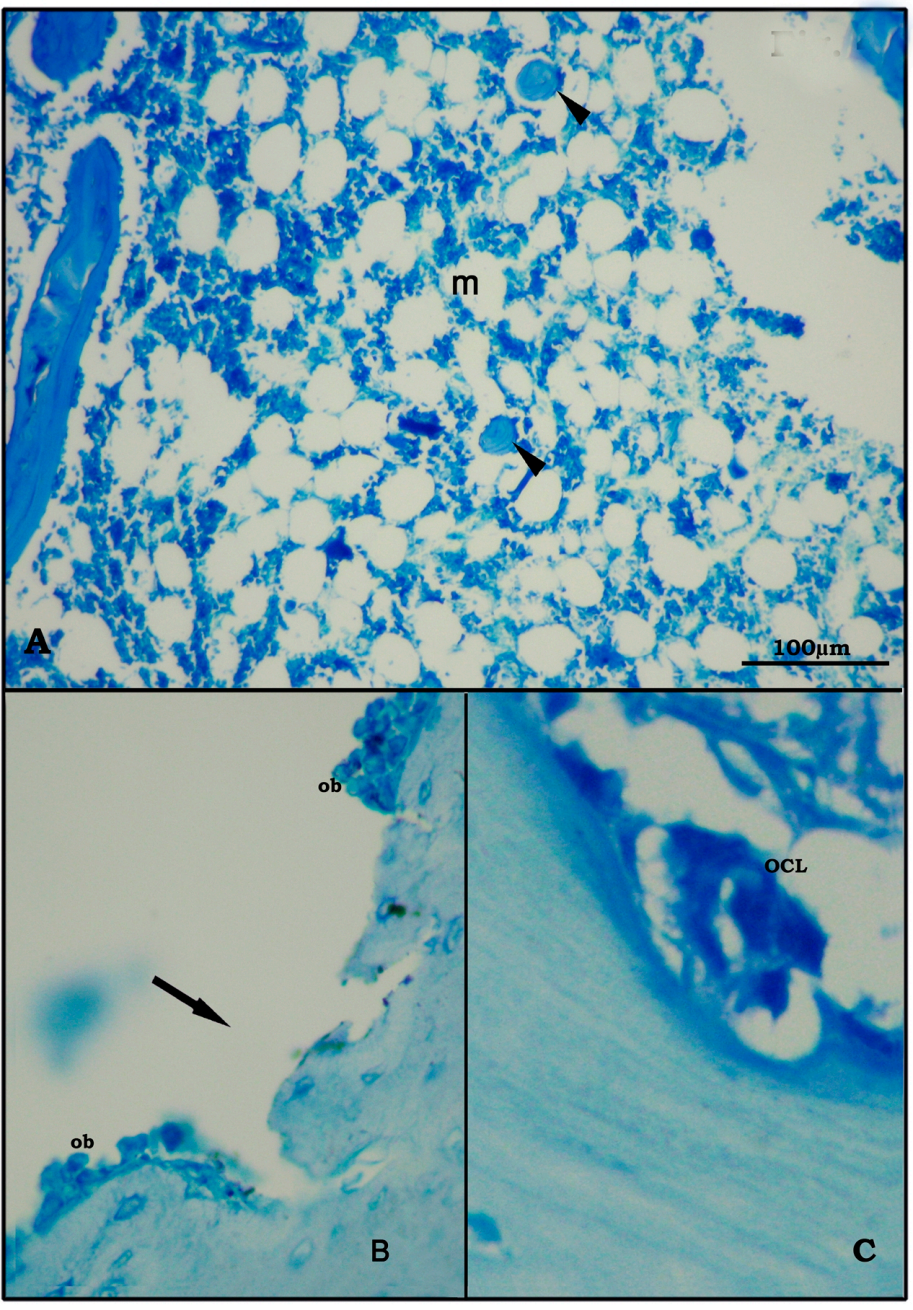
**Proximal metaphysis of tibia in the OVX-group (Azure II-Methylene blue)**. A: trabeculae (black triangle) of cancellous bone as separate bony ossicles with widened bone marrow spaces (m). (×250) B: eroded surface of a trabecula (↑) with proliferation of osteoblasts (ob) on sides of erosion. (×400) C: oscteoclast (OCL) in Howship's lacuna (×640).

Bone sections in the OVX-rats revealed thinning of the outer cortical bone as compared to the control group. The inner cancellous bone trabeculae lost their normal architecture and appeared as discontinuous bony ossicles separated by widened bone marrow spaces (Figure 3A, 4A). Osteoclasts were apparently increased as compared to the SHAM- operated control group (Figure 3B, C, 4C). Erosion cavities were detected in the endosteal surface in some trabeculae and proliferation of osteoblasts was also detected in some areas (Figure 4B). The Mean CBT and the mean TBT showed a significant decrease as compared to the SHAM- operated control group whereas the osteoclast number was significantly increased (Table 3).

**Table 3:** Cortical bone thickness (CBT), trabecular bone thickness (TBT) and osteoclast (OCL) number in: SHAM-operated control (SHAM) rats, Ovariectomized (OVX) rats, and Olive-supplemented ovariectomized (Olive-OVX) rats.

	SHAM	OVX	Olive-OVX
CBT (μm)	248.72 ± 3.69 n = 5	203.77 ± 2.07**^a, b^** n = 5	243.21 ± 2.64 n = 5
TBT (μm)	92.38 ± 1.15 n = 5	53.54 ± 1.71 **^a, b^** n = 5	86.11 ± 1.48**^a^** n = 5
OCL/10 HPF	6 ± 0.51 n = 5	14 ± 0.66**^a, b^** n = 5	7 ± 0.37 n = 5

n; is the number of observations

(a) Significance calculated by least significant difference (LSD), P < 0.05 from SHAM-operated control group.

(b) Significance calculated by LSD, P < 0.05 between OVX and Olive-OVX groups.

**Figure 5:**
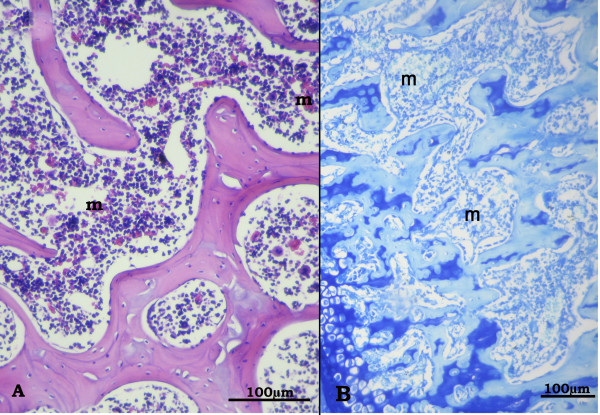
**Proximal metaphysis of tibia in the Olive-OVX group showing preserved architecture of trabeculae in comparison to the OVX-rats**. Trabeculae appear more or less continuous with bone marrow spaces (m) in between. A: H&E (×250), B: Azure II-Methylene blue (×200).

Sections in the metaphysis of tibia in the Olive-OVX rats revealed marked improvement as compared to those of the OVX-rats. The cortical bone thickness was very similar to the SHAM- operated control group. The cancellous bone trabeculae partially regained near normal structure and appeared more continuous with less widened bone marrow spaces (Figure 5). The mean CBT showed a non-significant change as compared to the SHAM- operated control group. On the other hand, the mean TBT showed a significant increase as compared to the OVX-rat group but was still significantly decreased as compared to the SHAM- operated control group. Meanwhile, the osteoclast number was non-significant from the SHAM- operated control group (Table 3).

### Liver

**Figure 6:**
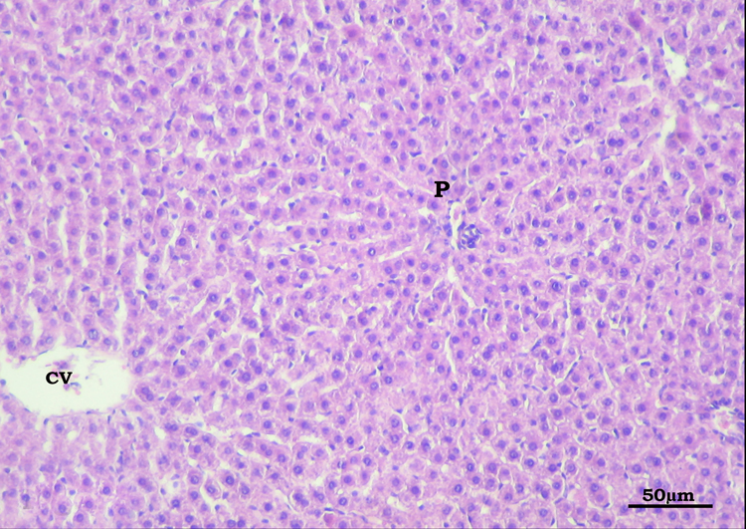
**Section in the liver of the control group showing branching and anastomosing cords of hepatocytes radiating from the central vein (CV) towards the portal area (P)**. Hepatocytes are separated by blood sinusoids lined by flat endothelial cells and Kupffer cells. (H&E X400)

Examination of sections of the livers of the SHAM-operated control group revealed that the parenchyma was formed of classic hepatic lobules having the central veins in the middle and the portal tracts at the periphery. From the central vein, branching and anastomosing cords of hepatocytes radiate. The hepatocytes appeared polyhyderal in shape, with mildly vacuolated cytoplasm and rounded vesicular nuclei. Blood sinusoids were situated between the cords of hepatocytes and lined by flattened endothelial cells and von Kupffer cells (Figure 6).

**Figure 7:**
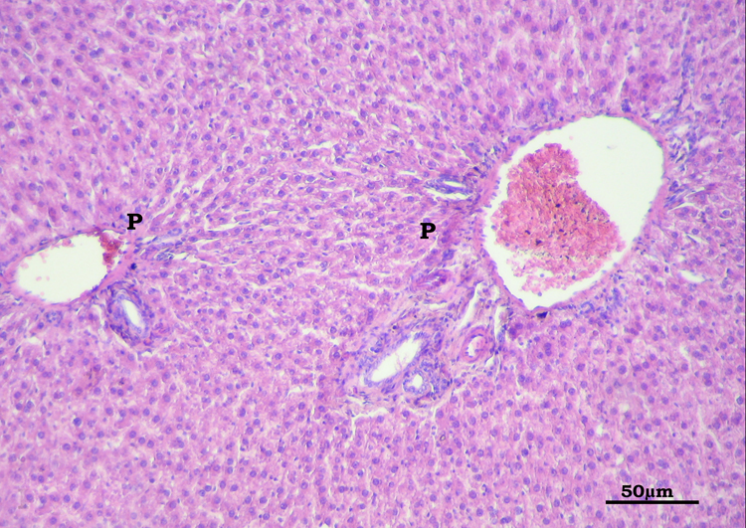
**Section in the liver of the OVX-group showing mononuclear cellular infiltration in the portal area(P)**. Notice the marked congestion of the blood vessels in the portal area. (H&E X400).

**Figure 8:**
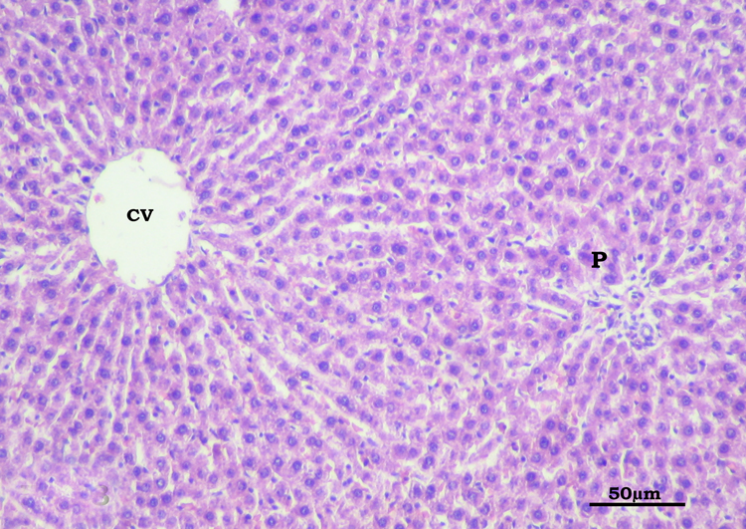
**Section in the liver of the Olive-OVX group showing a picture similar to the control**. Mononuclear cellular infiltration and congestion are not detected in the portal area (P) (CV: central vein). (H&E X400).

In the OVX-rats, the most notable finding was mononuclear cellular infiltration in the portal areas. Congested, dilated and thickened blood vessels were also detected in the portal areas (Figure 7). In the Olive-OVX rats, the structure was similar to the SHAM- operated control group. Mononuclear cellular infiltration and congestion were not detected (Figure 8).

## Discussion and Conclusions

The present study describes the effect of olive oil supplementation on osteoporosis induced by ovariectomy. In the current study, plasma alkaline phosphatase was significantly increased in the OVX-rats, indicating an increase in bone formation. Meanwhile, the increased number of osteoclasts and the erosion cavities detected by histological examination indicated that increased bone resorption was the major factor underlying the osteoporosis observed in the OVX-rats. Our findings were in agreement with former studies that have linked estrogen deficiency to acceleration of bone remodeling where osteoclastic bone resorption outpaced the anabolic activity of osteoblasts [11,12].

In the current study, histological examination of the liver of the OVX-rats showed mononuclear cellular infiltration. This finding is consistent with a previous experimental study showing that OVX mice display early hepatic inflammation [13]. Osteoporosis is a common complication of many types of liver disease. The production of cytokines including TNF, CSF1 and IL-17 can increase osteoclastogenesis and bone loss in inflammatory liver conditions [14]. The bone-protective effects of estrogen may involve suppression of inflammatory cytokines such as interleukin-1 (IL-1) and tumor necrosis factor-alpha (TNF-α), which in turn activate inducible nitric oxide synthase (iNOS). iNOS is only expressed in response to inflammatory stimuli, and nitric oxide (NO) derived from this pathway potentiates cytokine and inflammatory-induced bone loss [15]. This gives a possible explanation for the detected significant increase in the plasma nitrates level present in the OVX-rats in our study. Furthermore, a previous study reported that IL-1, and TNF-α, were increased in healthy premenopausal women who underwent ovariectomy and reached the highest levels 8 weeks after ovariectomy, and these changes in the cytokine profile were associated with indices of bone resorption [3].

In the current study, malondialdehyde (MDA) was significantly increased in the ovariectomized rats indicating increased oxidative stress in these estrogen-deficient rats. It has been demonstrated that free radicals intervene in bone resorption, promoting osteoclastic differentiation in such a manner that bone resorption is increased with oxidative stress [16]. The increased oxidative stress could be attributed to the loss of the antioxidant effects of estrogen [3]. Enhanced osteoclastic activity/number detected in our study may have been responsible for increased production of reactive oxygen species [ROS]. One the most important damaging effects of ROS on tissues is lipid peroxidation. Lipid peroxidation can be evaluated by the measurement of malondialdehyde (MDA) levels, which also served as a measure for osteoclast activity [17]. Thus, the observed increase of oxidative stress could have contributed to the osteoporosis observed in the OVX- rats.

In addition to estrogen, calcium metabolism plays a significant role in bone turnover, and deficiency of calcium leads to impaired bone deposition. Our data showed that ovariectomy led to significant hypocalcemia in the OVX-rats. A previous study reported that ovariectomy in rats resulted in an impaired calcium balance which could also have contributed to ovariectomy-induced osteoporosis [18]. Furthermore, menopause is associated with decreased intestinal calcium absorption [19]. The reduced calcium absorption has been attributed to reduced circulating 1,25-dihydroxyvitamin D levels, and to gastrointestinal resistance to the action of 1,25-dihydroxyvitamin D [20]. It has been shown in humans that oestrogens modulate the end organ effect of 1, 25-dihydroxyvitamin D on intestinal calcium absorption [21]. Also, menopause is associated with increased renal excretion of calcium [17]. Estradiol acts on the kidney to increase renal tubular reabsorption of calcium [22]. Accordingly, changes in estradiol levels are associated with changes in expression of many proteins involved in distal tubule calcium reabsorption [23].

In the current study, olive oil was effective in preventing ovariectomy-induced hypocalcemia in the Olive-OVX rats. Olive oil enhances intestinal absorption of calcium [24]. Moreover, olive oil is an excellent source of gamma linolenic acid (GLA), which has been shown to reduce the excretion of calcium, inhibit bone reabsorption and markers of bone turnover while at the same time increases the calcium content in the bone [25].

The current study revealed that olive oil supplementation to the OVX-rats attenuated ovariectomy-induced osteoporosis. One likely reason for this improvement in bone loss could be attributed to its high content of monounsaturated fatty acid, which has been reported to affect bone mineral density (BMD) [26].

Studies on olive oil support that bone mineralization needs fat, and that the best results are obtained when a minimum amount of polyunsaturates is added to oleic glycerides. This beneficial ratio is taken from diets consuming extra virgin olive oil [27].

Olive oil contains a group of related natural products with potent anti-inflammatory and anti- oxidant properties. Moreover, olive oil contains a healthy balance of omega-6 to omega-3 FFAs, which results in low inflammation [28,29]. Olive oil has been reported to decrease both oxidative stress and the production of arachidonic acid metabolites by the prostaglandin G/H synthase pathway in rat macrophages [30]. In agreement, our study clearly demonstrated reduced inflammatory infiltration of the liver, as well as reduced oxidative stress as indicated by the significant decrease in MDA levels in the Olive-OVX rats. Moreover, Olive oil has been reported to suppress the increase in NO production and activity of iNOS in rats [31]. In agreement, in the current study olive oil supplementation decreased plasma nitrate levels in the Olive-OVX rats. In conclusion; the anti-inflammatory and the anti-oxidant properties, as well as the blood calcium raising effects of olive oil prevented bone loss and decreased resorption of bone in the olive oil supplemented ovariectomized-rats. This suggests that olive oil represents a promising therapeutic option for the prevention and/or treatment of postmenopausal osteoporosis.

## Competing interests

The authors declare that they have no competing interests.

## Authors' contributions

NS carried out the practical part, participated in the design of the study, participated in the sequence alignment and drafted the manuscript and performed the statistical studies. HS carried out the histological and morphometric study, participated in sequence alignment, drafting and revision of manuscript. All authors read and approved the final manuscript.

## Pre-publication history

The pre-publication history for this paper can be accessed here:

http://www.biomedcentral.com/1472-6882/11/10/prepub
